# Time structures of proton pencil beam scanning delivery on a microsecond scale measured with a pixelated semiconductor detector Timepix3

**DOI:** 10.1002/acm2.14486

**Published:** 2024-08-13

**Authors:** Jiajian Shen, Xuanfeng Ding, Serdar Charyyev, Xiaoying Liang, Cristina Oancea, Peilong Wang, William G. Rule, Wei Liu, Martin Bues, Liyong Lin

**Affiliations:** ^1^ Department of Radiation Oncology Mayo Clinic Phoenix Arizona USA; ^2^ Department of Radiation Oncology Corewell Health Beaumont University Hospital Royal Oak Michigan USA; ^3^ Department of Radiation Oncology Stanford University Palo Alto California USA; ^4^ Department of Radiation Oncology Mayo Clinic Jacksonville Florida USA; ^5^ ADVACAM Prague Czech Republic; ^6^ Department of Radiation Oncology and Winship Cancer Institute Emory University Atlanta Georgia USA

**Keywords:** dose rate, pencil beam scanning, proton therapy, scanning speeds, semiconductor detectors, time structure

## Abstract

**Purpose:**

The time structures of proton spot delivery in proton pencil beam scanning (PBS) radiation therapy are essential in many clinical applications. This study aims to characterize the time structures of proton PBS delivered by both synchrotron and synchrocyclotron accelerators using a non‐invasive technique based on scattered particle tracking.

**Methods:**

A pixelated semiconductor detector, AdvaPIX‐Timepix3, with a temporal resolution of 1.56 ns, was employed to measure time of arrival of secondary particles generated by a proton beam. The detector was placed laterally to the high‐flux area of the beam in order to allow for single particle detection and not interfere with the treatment. The detector recorded counts of radiation events, their deposited energy and the timestamp associated with the single events. Individual recorded events and their temporal characteristics were used to analyze beam time structures, including energy layer switch time, magnet switch time, spot switch time, and the scanning speeds in the *x* and *y* directions. All the measurements were repeated 30 times on three dates, reducing statistical uncertainty.

**Results:**

The uncertainty of the measured energy layer switch times, magnet switch time, and the spot switch time were all within 1% of average values. The scanning speeds uncertainties were within 1.5% and are more precise than previously reported results. The measurements also revealed continuous sub‐milliseconds proton spills at a low dose rate for the synchrotron accelerator and radiofrequency pulses at 7 µs and 1 ms repetition time for the synchrocyclotron accelerator.

**Conclusion:**

The AdvaPIX‐Timepix3 detector can be used to directly measure and monitor time structures on microseconds scale of the PBS proton beam delivery. This method yielded results with high precision and is completely independent of the machine log files.

## 1 INTRODUCTION

The time structure characteristics of proton spot delivery in proton pencil beam scanning (PBS) radiation therapy are essential in many clinical applications. Specifically, understanding the time structures of proton beams are needed to calculate 4D structure doses to evaluate interplay effects,^1^, ^2^, ^3^, ^4^, ^5^, ^6^, ^7^, ^8^, ^9^ quantify the dose rates in FLASH proton therapy,^10^ and optimize proton plans using arc therapy.^11^ The micro time structures are essential for calculating the FLASH dose rate for pulse accelerator and further optimization.^12^, ^13^, ^14^

In a previous publication,^15^ we modelled the time structures of the Hitachi ProbeatV5 PBS system (Hitachi, Ltd, Tokyo, Japan). The method proposed by Shen et al.^15^ used specifically designed spot delivery patterns and associated beam delivery log files to derive the key elements in the time sequence of beam delivery, including times for energy switch, spot delivery preparation, spot MU delivery, and moving to the next spot position. The experimental method was later implemented to model the time structures of two ion beam applications (IBA, Louvain la Neuve, Belgium) proton systems: Proteus®One^16^ and ProteusPlus.^17^

While the aforementioned approach proved to be effective in certain proton PBS systems, it is an indirect method and relies on machine log files. Its implementation becomes more challenging for more complex system, such as the new Hitachi machine with multiple energy extractions^18^, ^19^ and dose‐driven continuous delivery.^20^ Moreover, the temporal resolution in the machine log files are insufficient to meet the requirements of FLASH therapy, making the above method impractical for FLASH therapy.

Recently, a direct method^21^ using two pixelated semiconductor detectors with Timepix3 chips (ADVACM s.r.o., Czech Republic) was applied to measure the time structures of the ProBeam (Varian Medical Systems, Palo Alto, CA) proton beam delivery system. The detectors with Timepix3 chips can achieve a high time resolution of 1.56 ns for individual particles,^22^, ^23^ offering a significant advantages in measuring the time structures of proton delivery systems.

The types of accelerators for proton beam therapy systems are usually cyclotron, synchrotron, and synchrocyclotron, which have different time structures for beam delivery. So far, the direct measurement method using semiconductor detectors^21^ was only applied to ProBeam systems, which use a cyclotron accelerator. The purpose of this study is to extend the direct method of using the high temporal resolution detector, AdvaPIX‐Timepix3, to measure and characterize the beam delivery time structures of the synchrotron and synchrocyclotron accelerators. Moreover, in this investigation, the method is significantly simplified compared to the prior work by Charyyev et al.^21^ In their study, two detectors were employed to measure prompt gamma rays and secondary neutrons, demanding sophisticated skills for post‐processing raw data to differentiate the particles. In contrast, our study only utilizes the raw data of the time of arrival (ToA) of all detected events, simplifying the process.

## 2 METHODS AND MATERIALS

### 2.1 AdvaPIX‐Timepix3 detector

AdvaPIX‐Timepix3 (ADVACM s.r.o., Czech Republic) is a fast, spectral imaging camera with an advanced semiconductor pixel application specific integrated circuit (ASIC) readout chip, Timepix3, bonded to 500 µm thick silicon sensor.^23^ It has a fast readout speed of 4 × 10^7^ hits/s, and a sensitive area of 14.08 mm × 14.08 mm with a high spatial resolution of 55 µm. For each ionizing particle, it digitally registers its position, energy loss, ToA, and track shape.^24^ In this work, the detector was operated in data‐driven mode (pixels) where two signal channels per pixel were set to register simultaneously ToA and *deposited energy* of individual particles reaching the sensor at the pixel level. The ToA can be identified with a resolution of 1.56 ns, whereas the Time over Threshold (ToT) of the respective pixel, and consequently the deposited energy, can be measured with energy resolution in orders of several keV.^25^ This detector was calibrated to a minimum energy threshold of 3 keV set during the per‐pixel energy calibration processed performed at Advacam Laboratory.^26^ The principle of the presented method is based on the fact that each radiation hit on the detector generated energy deposition in multiple pixels, and the counter of the energy deposition events was continuously incremented if the deposited energy is larger than the threshold energy of 3 keV. The data were saved in text files, which include the counter and the timestamp of the energy deposition. Other Digital‐to‐Analog Converter (DAC) parameters were not changed from their standard values except the bias voltage which was set at +80 V, a typical value for proton particle tracking.

A more detailed description of the detector and its applications in proton PBS can be found in other papers.^21^, ^22^, ^23^, ^27^, ^28^, ^29^, ^30^ However, for the aim of this work, is needed only the timestamp of each event registered by the detector, and the high temporal resolution is sufficient for detecting the beam on and off times accurately.

### 2.2 Experimental setup

**FIGURE 1:**
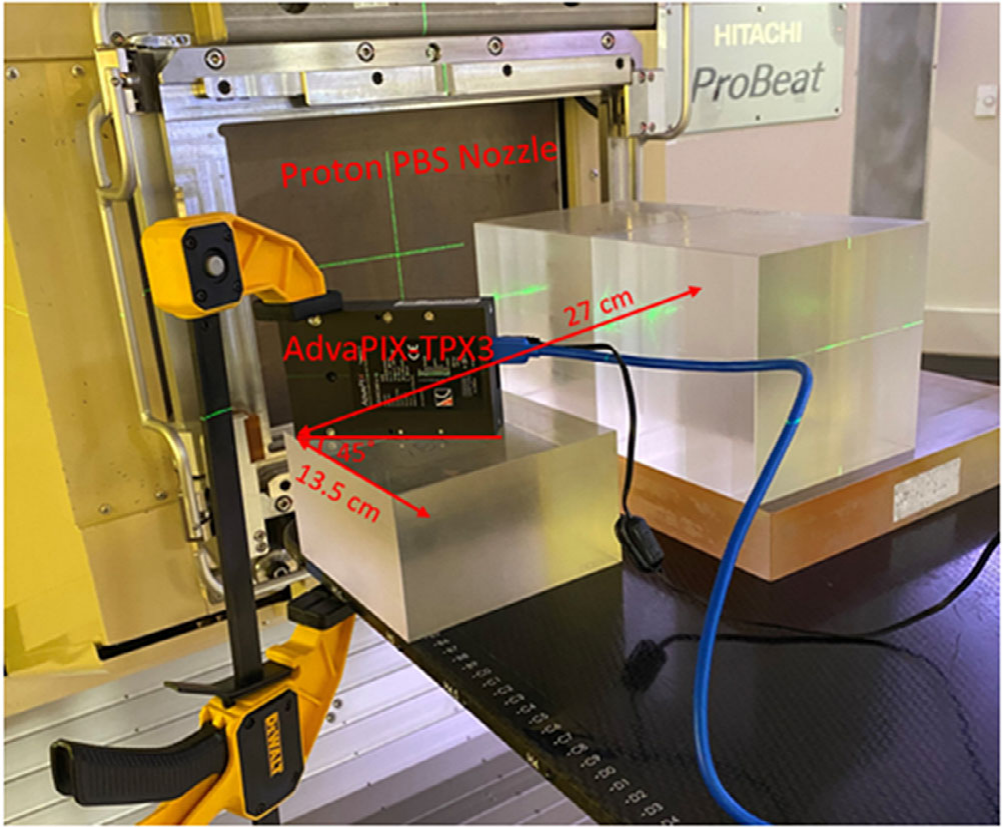
Experimental setup inside the treatment room. Plastic blocks were aligned to the central axis of the beam to stop protons. The AdvaPIX‐Timepix3 detector was positioned laterally at an angle of 45° to the central axis, with the furthest edge at 27 cm lateral and 13.5 cm upstream to the isocenter. A clamp was used to stabilize the detector.

The experiment was carried out in a treatment room with gantry inclined at 90° as illustrated in Figure 1. Acrylic blocks (33 cm water equivalent thickness) were used to stop the protons of 228.8 MeV energy. The AdvaPIX‐Timepix3 detector was fixed (using a clamp) laterally at a scattering angle of 45°, and the furthest edge of the detector was positioned at 27 cm laterally and 13.5 cm upstream to isocenter, respectively. In this experimental setup, the field‐of‐view detects mostly scattered particles from beam nozzle and the prompt gamma generated by the primary particles. Passive cooling (fan) was used to maintain a constant temperature of less than 25°C at detector's sensor. The AdvaPIX‐Timepix3 detector was connected to a laptop via a USB cable and operated using the PIXET PRO (V1.8.0) software provided by the manufacturer.

### 2.3 Accelerator and proton beams used in the experimental setup

The experiments were conducted using a Hitachi ProBeatV5 PBS system. A synchrotron‐based proton beam therapy system featuring 97 discrete energies ranging from 71.3 to 228.8 MeV was used in the experimental setup from Figure 1. The minimum MU for this system is 0.003 MU, corresponding to approximately 3 × 10^6^ protons at an energy of 228.8 MeV.^31^ The system utilized discrete spot delivery, resulting in a beam off time between consecutive spots. This beam off time included the time for the magnet switch (about 2 ms) and the time for moving from the current spot position to the next spot position. The proton spill for each spot occurred at a low beam current of about 1.0 nA and fluctuated by approximately 10%.^15^ Additionally, there was a much longer energy layer switch time of about 2 s. To acquire precise timings for energy switches, magnet switches, and scanning speeds for moving spots in both *x* and *y* directions, multiple specially designed beams were created, each serving a distinct measurement purpose. The description of the designed beam and how to use them to measure the time structures are included below.

To measure the energy switching time (*T_ESw_*), four single energy beams were generated for E = 71.3, 140.2, 200.4, and 22.8 MeV, each consisted of 10 spots directed at isocenter (*x* = 0, *y* = 0) with the minimum MU (0.003) for each spot. While 71.3, 140.2, and 228.8 MeV represented the low, medium, and high energies, an additional energy of 200.4 MeV was also specifically included due to its longer *T_ESw_*, serving as an outlier among the 97 energies.^15^ Each beam was repeated 30 times to reduce measurement uncertainty. The *T_ESw_* can be defined as the time interval between two consecutive energy layers, that is, from the timestamp of the last event in the prior energy layer to the timestamp of the starting event in the next energy layer.

To measure the magnet switch time (*T_MSw_*), a beam with a single energy layer and 100 spots was generated. All spots were directed at the isocenter (*x* = 0, *y* = 0) with the minimum MU (0.003). In the case of this specially designed beam, where all spots occupy the same location, there is no moving time between consecutive spots. Consequently, the *T_MSw_* was defined as the time interval between the timestamp of the last event in the prior spot and the timestamp of the starting event in the next spot. Since *T_MSw_* did not depend on beam energy,^15^ only energy 228.8 MeV was used to obtain *T_MSw_*.

To measure the scanning speeds (m/s) in the *x* (*V_x_*) and *y* (*V_y_*) directions, a beam with a single energy layer and 100 spots, each with 0.003 MU, was generated. For *V_x_*, spot positions alternated between (*x* = −5 cm, *y* = 0) and (*x* = 5 cm, *y* = 0), creating a moving distance of 10 cm in the *x* direction for consecutive spots. Similarly, for *V_y_*, spot positions alternated between (*x* = 0, *y* = −5 cm) and (*x* = 0, *y* = 5 cm). The spot switch time (*T_SSw_*) was defined as the time interval between two consecutive spots, that is, the timestamp of the last event in the prior spot to the timestamp of the starting event in the next spot. The spot switch time is the sum of the *T_MSw_* and time to move the spot to the next position. Consequently, the scanning speed can be derived using the formula below:

(1) $$V\left(m/s\right)=\frac{\Delta L}{\Delta T}=\frac{0.1\;\left(m\right)}{\bar{T_{SSw}}\;-\;\bar{T_{MSw}}}$$

where the average bar in Equation (1) represents the average value from multiple measurements of *T_SSw_* and *T_MSw_*. This test beam was delivered separately for three different energies covering the representative low, medium, and high energies at 71.3, 140.2, and 228.8 MeV, respectively. A theoretical curve that shows how the scanning speeds vary with the proton energies was fitted to the measured scanning speeds. For protons with a given kinetic energy *E (MeV)*, the scanning speeds *V_x_* and *V_y_* follow the equations below^20^:

(2) $$v_{x,y}^{E}=v_{x,y}^{E_{max}}\times \frac{\sqrt{E_{max}^{2}+2\times E_{max}\times E_{0}}}{\sqrt{E^{2}+2\times E\times E_{0}}}$$

where, $v_{x,y}^{E_{max}}$ is the scanning speed in *x* or *y* direction for the maximum proton energy *E_max_* (i.e., 228.8 MeV in our system) and $E_{0}$ is the rest energy of a proton (938 MeV).

### 2.4 Uncertainty analysis

To validate the accuracy of the directly measured time in this work, we designed a specific beam with a single spot at 0.1 MU and 228.8 MeV. This beam delivery was repeated 10 times. We then compared the directly measured beam delivery time to the time recorded by the machine log file.

For each measured parameter in PBS time structures, repeated 30−100 times measurements were taken on the same day to calculate the average value and standard deviation. All measurements were repeated on three different dates. The final reported measurement uncertainties were the averages and the standard deviations of the average values from three measurement days.

The measured parameters were also compared to the values provided by the proton beam therapy delivery system vendor, Hitachi, and the derived values from a prior work.^15^

### 2.5 Feasibility test for the synchrocyclotron accelerator

The same detection system was implemented to William Beaumont proton center, which houses an IBA Proteus®One PBS system equipped with a compact super conducting synchrocyclotron accelerator. The time structures of the beam at this facility differs from both a cyclotron and a synchrotron system. It has the unique characteristics of high‐intensity pulsed beam and splits each layer into several radiation bursts. A spread‐out Bragg peak (SOBP) beam was delivered with an experimental setup similar to that shown in Figure 1. The purpose of this part of the experiment was to demonstrate the feasibility of the detector to capture the unique time structures for this type of PBS system, including the burst switch time, the high‐intensity pulse in microseconds, and the dead time between consecutive pulses in milliseconds.^16^

## 3 RESULTS

### 3.1 Time gaps used for measuring the key parameters of the time structures

**FIGURE 2:**
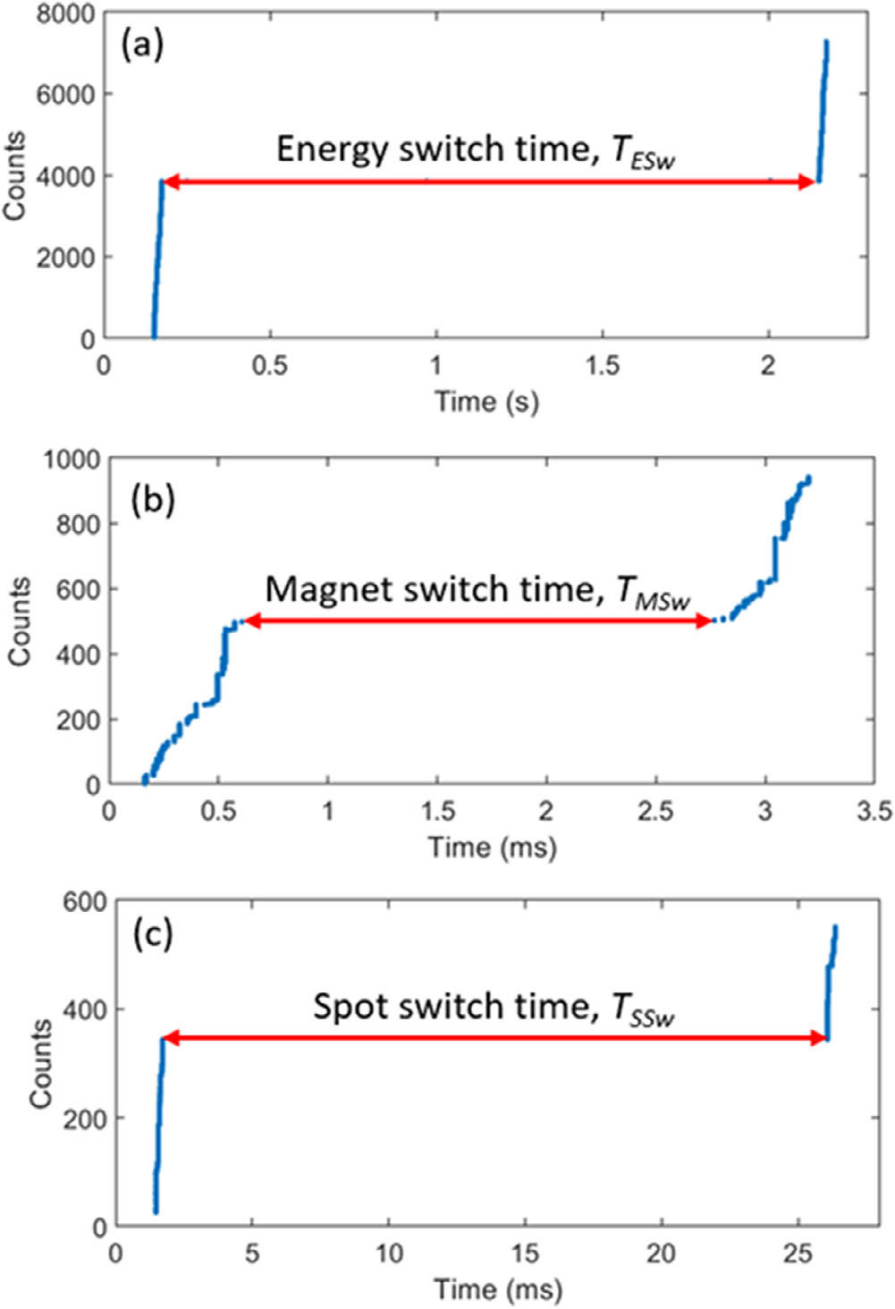
Sample beam structures to measure the energy switch time (a), magnet switch time (b), and the spot switch time (c). The blue curves depict the raw data of pixel counts of two consecutive spots.

Figure 2 shows the sample time structures measured directly by the AdvaPIX‐Timepix3 detector, where the *y* axis represents the counts of pixel hits registered by the detector, and the *x* axis is the timestamp associated with the corresponding counts. The energy switch time, the magnet switch time, and the spot switch time are shown in the subfigures (a)–(c), respectively. In each subfigure, the blue curves represent the counts from consecutive spots. In (a), the next spot occurred after an energy switch; in (b), the two spots were both delivered to the isocenter in the same energy layer, and in (c), the two spots were in the same energy layer but had a spot spacing of 10 cm in the *x*‐direction. The spot switch time includes both the magnet switch time and the scanning time to move the spots to the next position. In Figure 2(b) it clearly showed sub‐milliseconds continuous proton spill.

As shown in Figure 2(b), there are about 500 counts in 0.5 ms, so the hit rate to the detector is about 1 × 10^6^ hits/s, which is well within the maximum readout speed of 4 × 10^7^ hits/s. Therefore, there is no need to worry about the pulse pile‐up due to too much radiation hits the detector simultaneously.

### 3.2 Comparison of measured beam delivery time to log file

The average measured beam delivery time from the 10 repeated beams was 12.28 ms, compared to 12.30 ms recorded in the log file. The average deviation was less than 0.02 ms, or 0.15%, with a deviation range of ±0.15 ms (±1.2%) across the 10 measurements.

### 3.3 Energy layer switch time

The measured *T_ESw_* are shown in Table 1, and they are consistent within 0.02 s (1%) with the prior work. Additionally, it is observed, consistent with the prior work, that the *T_ESw_* for E = 200.4 MeV took a longer time than the other energies.

**TABLE 1:** The nominal and experimentally determined parameters for time structures of Hitachi ProbeatV5 system.

Parameter	Nominal values^a^	Experimental values	Prior work^b^
*T_ESw_* (s)	1.905	1.991 ± 0.013; E = 228.8 MeV	2.00; E = 228.8 MeV
		2.077 ± 0.001; E = 200.4 MeV	2.05; E = 200.4 MeV
		1.915 ± 0.036; E = 140.2 MeV	1.90; E = 140.2 MeV
		1.888 ± 0.033; E = 71.3 MeV	1.90; E = 71.3 MeV
*T_MSw_* (ms)	2.2	2.103 ± 0.017	1.93 ± 0.02
		24.27 ± 0.01; E = 228.8 MeV	
*T_SSw_* (ms, x)		19.07 ± 0.01; E = 140.2 MeV	
		13.95 ± 0.04; E = 71.3 MeV	
		8.84 ± 0.03; E = 228.8 MeV	
*T_SSw_* (ms, y)		7.25 ± 0.03; E = 140.2 MeV	
		5.69 ± 0.04; E = 71.3 MeV	
	6	4.51 ± 0.06; E = 228.8 MeV	5.0; high E group
***V_x_*** (m/s)		5.89 ± 0.08; E = 140.2 MeV	5.7; med E group
		8.44 ± 0.12; E = 71.3 MeV	7.0; low E group
	10	14.84 ± 0.19; E = 228.8 MeV	17.1; high E group
***V_y_*** (m/s)		19.43 ± 0.25; E = 140.2 MeV	18.2; med E group
		27.85 ± 0.37; E = 71.3 MeV	22.2; low E group

^a^ Nominal values are provided by the vendor of the accelerator.

^b^ Values extracted from Table 2 of a prior work.^15^

### 3.4 Magnet switch time

The measured *T_MSw_* in this work is 2.103 ± 0.017 ms (i.e., 0.8% uncertainty). When compared to the value of 1.93 ms from prior work, it is closer to the nominal value (2.2 ms) provided by the vendor.

### 3.5 Magnetic scanning speeds

**FIGURE 3:**
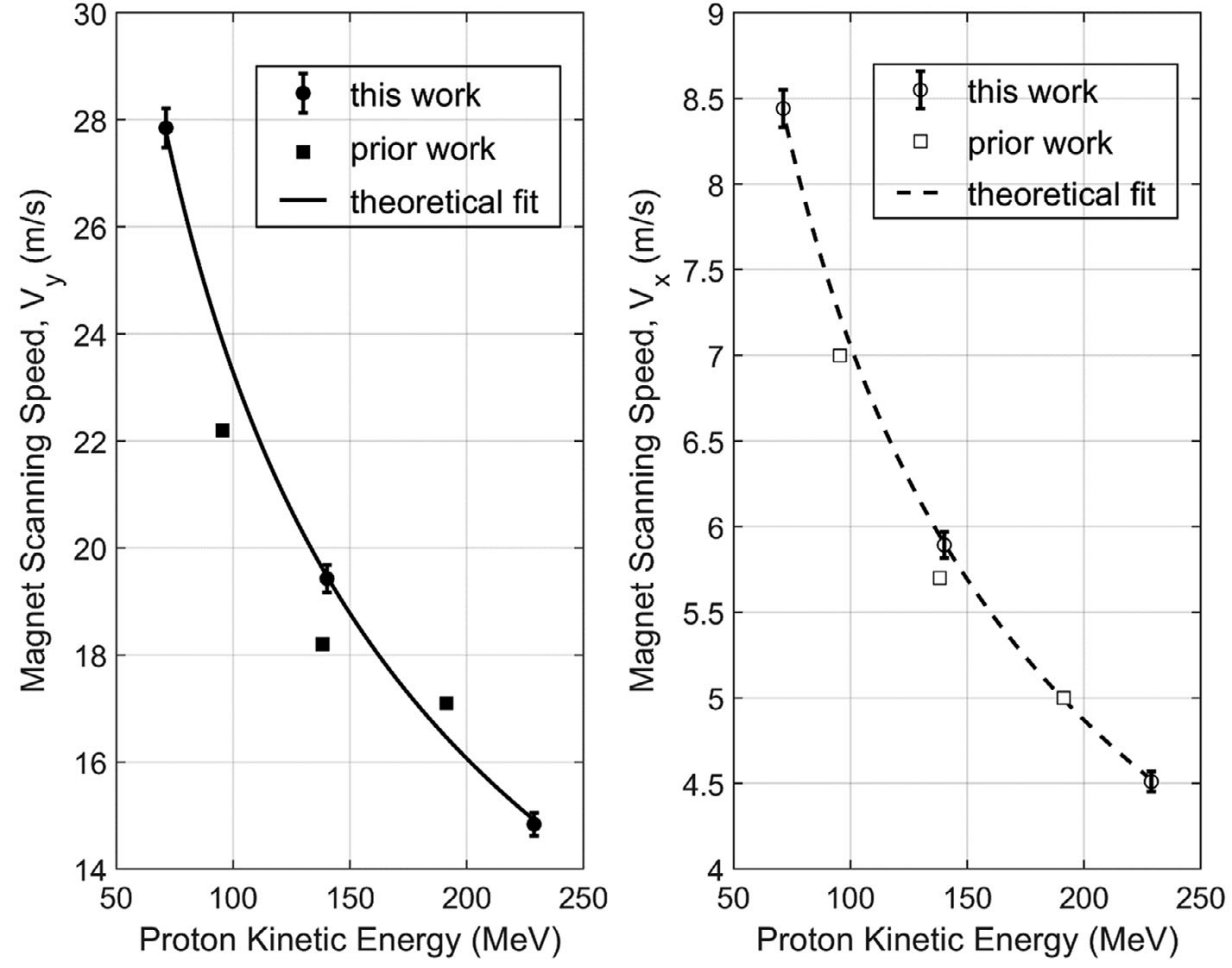
Scanning speeds in the *y* and *x* directions are shown in the left and right panel, respectively. The measured scanning speeds in the *y* and *x* directions are represented by the filled and open circle symbols, respectively. The error bars accompanying the measured scanning speeds indicate the level of uncertainties in the measurements. The smooth curves represent the best fit to the measured scanning speeds using Equation (2). For comparison, the scanning speeds reported in a prior work^15^ are shown using square symbols.

The measured *V_y_* and *V_x_* from this work are shown in the left and right panels in Figure 3 as the filled and open circle symbols, respectively. The error bars represent the measurement uncertainty for each scanning speed and are based on the error propagation from Equation (1). Thus, it includes the spot position uncertainty, which is 1 mm in our system. For the spot moving distance of 10 cm, it contributes to 1% of the scanning speed uncertainty. The uncertainty of the *T_MSw_* is 0.8%. The uncertainty of the *T_SSw_* varies with proton energies and scanning directions. The protons with the lowest energy (71.3 MeV) in the *y* direction have the largest uncertainty of 0.7% because they have the fastest scanning speed and *T_SSw_* is only 5.7 ms. On the contrary, the uncertainty of the highest energy (228.8 MeV) in the *x* direction is only 0.06%, and *T_SSw_* is 24.3 ms.

The smooth curves in Figure 3 represent the best fit to the measured scanning speeds using Equation (2). It shows that the measured scanning speeds match precisely with theoretical curves about how the scanning speeds change with the proton energies. As a comparison, the scanning speeds obtained from a prior work^15^ are also shown in Figure 3 as square symbols, which slightly deviated from the theoretical curves. For example, the previously measured *V_y_* at intermediate energy (18.2 m/s) is 6.2% lower than the theoretical curve. However, the direct measurements in this study perfectly match the theoretical fit. These comparisons manifest that the measured scanning speeds in this work have high precision, and the direct measurement methods are more preferable than the indirect methods.^15^

Since the specification of the spot position accuracy of our current proton PBS system is about 1 mm, the measured scanning speeds would have much larger uncertainty if the spot moving distance is too small. In this work, 10 cm was chosen to control the uncertainty to 1%. If a smaller spot moving distance, such as 1 cm, was chosen, the uncertainty would increase to 10%, significantly affecting the accuracies of the measured scanning speeds.

### 3.6 Time structures for IBA Proteus®One

**FIGURE 4:**
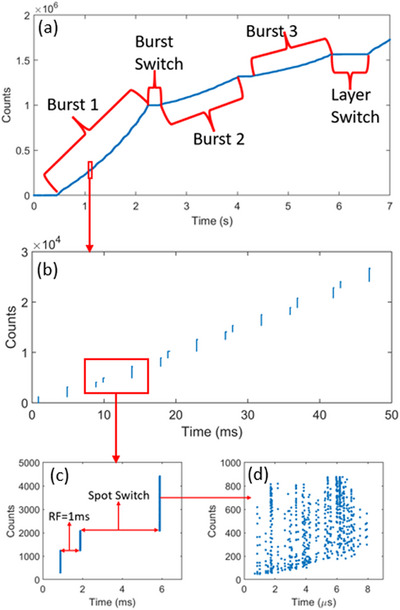
Time structures for the IBA Proteus®One machine. A selected beam structures in 7 s is shown in (a), which illustrates the three bursts in a given energy layer, the switch time between bursts, and the energy layer switch after the last burst of the layer. A zoom‐in of (a) in 50 ms window is shown in (b), which depicts the pulsed delivery for the spots. A further zoom‐in of (b) in a 7 ms window is shown in (c), which reveals the pulsed beam in a 1 ms repetition time, and the switch time to the next spot. The last zoom‐in of (c) is displayed in (d), which shows a high‐intensity pulse with a beam‐on time for about 7 µs.

The time structures for the IBA Proteus®One machine are shown in Figure 4. Figure 4(a) captured 7 s of the time structures from this machine. It shows that a given energy layer was split into three bursts, with burst 1 delivering most of MU in the layer. The beam then switched to the next layer after the last burst was delivered. The burst switch time and the layer switch time are about 0.22 and 0.73 s, respectively, which agrees with the previous finding using machine logfiles.^16^

To further explore the time structures in a finer time scale, a 50 ms zoom‐in window from Figure 4(a) is displayed in Figure 4(b). It shows the spot delivery is not continuous, which is significantly different from the continuous spot delivery from the Varian ProBeam machine.^21^ A further zoom‐in of a 7 ms window was selected from Figure 4(b) and displayed in Figure 4(c). It shows the pulsed beam delivery with 1 ms repetition time, and the time it takes to switch to the next spot. Figure 4(d) is the final zoom‐in to show the high‐intensity proton spill in a period of about 7 µs.

## 4 DISCUSSION

In this work, we demonstrated that the AdvaPIX‐Timepix3 detector is a convenient tool for measuring the time structures of therapeutic proton beams on various time scales. Compared to the indirect method,^15^ this direct method is straightforward, easy to implement, and independent of the log file. More importantly, it also yields results with higher precision, for example, the scanning velocities shown in Figure 3. In theory, as shown in Figure 2, the key parameters of the time structures can be extracted quickly with just a single capture of the events. However, it is recommended to take a larger number of samples to reduce statistical uncertainty. For example, the uncertainty of the individual *T_MSw_* is about 4%. However, by using the average of multiple measurements, the average ($\bar{T_{MSw}}$) at different dates is highly consistent within 0.8%.

The exact geometry shown in Figure 1 for placing the detector was primarily to ensure reproducibility of the setups on different days. However, we do not believe that a slight change in the detector's positioning would affect the results, as the detector has a wide dynamic range for measured events. The only factor to avoid is event pile‐up, that is, more than 4 × 10^7^ hits/s. This limit can be easily exceeded if the detector is placed directly in the proton beam's path, which could even damage the detector. Placing the detector laterally, more than 20 cm away from the proton beam, can effectively keep the hit evens well within the limit.

It is also worth mentioning that we initially followed the setup of Charyyev et al.^21^ placing the detector well beyond the distal end of the proton beam to measure the Gamma fluence. However, the measured events were too low for our proton system. We believe this is due to the low dose rate in our synchrotron system, whereas the dose rate in Charyyev et al.^21^ Varian ProBeam system can be 100–1000 times higher than ours. Therefore, for centers using a synchrotron‐system that only extracts protons at low dose rates, placing the detector laterally is more sensitive than placing it at the distal end after the Bragg peak.

Figure 4 shows the different time parameters of energy layer switch, burst switch, pulsed beam delivery with 1 ms repetition time, and even high‐intensity proton pulses in the order of microseconds can be detected precisely by AdvaPIX‐Timepix3. Figure 2(b) clearly shows a sub‐milliseconds proton spill for a single spot, and the protons delivered within the single spot by the synchrotron accelerator are nearly continuous. This continuous beam delivery within the spot is fundamentally different from the pulsed structure of the synchrocyclotron accelerator as shown in Figure 4(b) and (c).

We presented extensive data and a detailed analysis for the synchrotron‐based Hitachi ProbeatV5 system. We not only measured the time parameters precisely, but also analyzed the uncertainties associated with the measured parameters. However, for the synchrocyclotron‐based IBA Proteus®One system, we only demonstrated the feasibility of the method. The trip to William Beaumont proton center was short, and we did not get enough beam time to conduct sufficient measurements. Hence, this is a limitation of this study. Since the IBA Proteus®One system is widely used and is a major future product for IBA, a thorough investigation of the time structures for the synchrocyclotron‐based proton systems is warranted in a future study.

## 5 CONCLUSIONS

The AdvaPIX‐Timepix3 detector was used to directly measure the key timing parameters for energy layer switch, magnet switch, and spot switch. The scanning speeds in the *x* and *y* directions are derived using the measured key parameters. All the measurements made under the synchrotron‐based proton accelerator were repeated multiple times and on different dates to reduce statistical uncertainty. The feasibility of using this detector to explore time structures for synchrocyclotron‐based proton accelerator was also demonstrated. The measured timing parameters using this detector have higher precision than the previously published work. The measurements clearly showed sub‐milliseconds continuous proton spill with a low spill rate for synchrotron accelerator, and a high‐intensity microsecond pulse beam from the synchrocyclotron accelerator.

## AUTHOR CONTRIBUTIONS

**Jiajian Shen**: Conceptualization; methodology; data curation; writing—original draft. **Xuanfeng Ding**: Investigation; data curation; writing—review & editing. **Serdar Charyyev**: Methodology; validation; writing—review & editing. **Xiaoying Liang**: Methodology; validation; writing—review & editing. **Cristina Oancea**: Methodology; software; validation; writing—review & editing. **Peilong Wang**: Data curation; writing—review & editing. **William G. Rule**: Resources; writing—review & editing. **Wei Liu**: Methodology; writing—review & editing. **Martin Bues**: Methodology; writing—review & editing. **Liyong Lin**: Conceptualization; methodology; validation; resources; writing—review & editing.

## DISCLOSURE

Xiaoying Liang received IBA and Elekta Industry research grants and honorarium outside the work presented here.

## CONFLICT OF INTEREST STATEMENT

The authors declare no conflict of interest.
